# Health-related quality of life measured using the EQ-5D–5 L: population norms for the capital of Iran

**DOI:** 10.1186/s12955-020-01365-5

**Published:** 2020-04-25

**Authors:** Zahra Emrani, Ali Akbari Sari, Hojjat Zeraati, Alireza Olyaeemanesh, Rajabali Daroudi

**Affiliations:** 1 Department of Health Management and Economics, School of Public Health, Tehran University of Medical Sciences, Poursina Ave, Tehran, 1417613191 Iran; 2 Department of Epidemiology and Biostatistics, Tehran University of Medical Sciences, Tehran, Iran; 3 National Institute for Health Research & Health Equity Research Centre, Tehran University of Medical Sciences, Tehran, Iran

**Keywords:** EQ-5D, Health related quality of life, Utility score, Iran

## Abstract

**Objectives:**

EQ-5D is the most commonly used generic preference-based health-related quality of life (HRQoL) measure. The current study aimed at estimating the HRQoL index scores using EQ-5D-5 L measure in the capital of Iran; moreover, identifying some determinants of the HRQoL.

**Methods:**

A sample of 3060 subjects was selected by a stratified random sampling method from the general adult population of Tehran. Face-to-face interview was conducted to fill out the questionnaire, in this cross-sectional survey. EQ-5D-5 L utility score were estimated using an interim value set, based on a crosswalk methodology. Additionally, the relationships between HRQoL and sociodemographic characteristics were tested by generalized linear model, using STATA version 13.

**Results:**

The mean ± standard deviation utility and EQ-VAS scores were 0.79 ± 0.17 and 71.72 ± 19.37. The utility scores ranged 0.61 ± 0.19 in > 69 year-old females to 0.88 ± 0.12 in < 30 year-old males. In mobility, self-care, and usual activity dimensions, most of the respondents reported “no problems” (70.47, 90.62, and 76.34%, respectively). However, in anxiety/depression and pain/discomfort dimensions, most of the respondents had problems (53.23 and 54.03%, respectively). Females had lower utility score than males; the utility score reduced with age increase; the educational level lead to higher utility scores; and the utility scores of individuals without spouse (divorced or widowed) were lower than those of the married individuals and never married ones.

**Conclusions:**

The current study reported HRQoL norm data for the general adult population in the capital of Iran; these data could be very useful for policy making and economic evaluations. A significant percentage of people in Tehran reported anxiety/ depression, which highlights the risk of psychological problems. Effective interventions are needed to increase their HRQoL, especially for the vulnerable groups of the community.

## Introduction

Nowadays, Health-related quality of life (HRQoL) has become an important health outcome indicator, and it is considered a primary outcome in many clinical trials [1]. HRQoL focuses on factors that are part of the person’s health (well-being and functioning) [1]. Different instruments are developed to measure HRQoL. Some of them are disease specific (e g, St George’s asthma quality of life scale, NEWQOL-6D, EORTC QOL-30), while some are generic (e g, EQ-5D, SF-6D, WHOQOL) [2].

The EuroQol five-dimensional (EQ-5D) is a very popular generic and preference-based instrument to make index values and health profiles [1]. It is a multi-attribute instrument, which considers five dimensions including mobility, self-care, usual activities, pain/discomfort, and anxiety/depression. There are two versions of EQ-5D [3]. In three-level version 243 (3^5^) health states are derived from the sample to establish a utility weight system. Levels are “no problems, some problems, and severe problems”. Many studies reported measurement insensitivity [4] and ceiling effect [5–7] about EQ-5D-3 L questionnaire. These issues are resolved to some extent in the new version. In five level version of EQ-5D the levels changed to “no problems, slight problems, moderate problems, severe problems, and unable to do/extreme” [8–10].

In many countries, this questionnaire is applied to evaluate population health status for which population norms are derived. The utility scores are extracted by gender, age, and other sociodemographic characteristics [9, 11, 12]. These studies provide the requisitions for health status surveillance and wider economic evaluations.

Although the three level EQ-5D utility scores for Iran are calculated by a previous study [13], as yet the HRQoL scores of general population based on EQ-5D-5 L is not reported. The current study aimed at providing the HRQoL scores and their determinants among the adult population of Tehran. The results improve the knowledge of the policy makers about the HRQoL of Tehran population and characteristics associated with their general health states to facilitate decision making, and health economic evaluations studies in Iran.

## Materials and methods

The current cross-sectional observational study was conducted on 3060 individuals in Tehran (the capital city of Iran) with the minimum age of 18, from October 2015 to March 2016. They were selected by a stratified random sampling method. First, Tehran population was divided into 22 municipal districts. Each district was divided into several blocks. Some blocks were randomly selected in each district. Number of selected blocks was determined according to the population of each district, and 300 blocks were totally selected for data collection. In each block, 10 households were randomly selected for interview. The respondents were selected by quota sampling in proportion to their basic social-demographic characteristics (gender, age) to be a representative sample of the Tehran adult population according to the data from the most recent census (Table 1). The households with non-responses were replaced with households in the replacement list. The interviews were conducted face-to-face by some trained interviewers.

**Table 1:** Sociodemographic characteristics of the sample (*N* = 3060)

Variable		Study sample		Tehran adult population [14]
		n	%	%
**Gender**	Male	1506	49.2	49.8
	female	1555	50.8	50.2
**Age (year)**	< 30	609	19.8	31.7
	30–39	726	23.7	22.6
	40–49	602	19.7	18.2
	50–59	539	17.6	13.7
	60–69	389	12.7	7.7
	> 69	195	6.37	6.1
**Education level**	Illiterate	140	4.6	6.7
	Elementary	387	12.6	15.4
	Guidance	516	16.9	16.1
	High school and pre-university	1100	35.9	33.2
	University	917	30.0	28.5
**Employment status**	Employed	1079	35.3	38.7
	Student	221	7.2	7.6
	Homemaker	1239	40.5	32.1
	Retired	395	12.9	10.5
	Unemployed	100	3.3	5.9
	Others	22	0.7	5.3
**Marital status**	Never married	549	18.0	23.5
	Married	2358	77.3	68.6
	Divorce or widowed	144	4.7	8.0
**Experience of a serious illness**				
In self	Yes	319	10.4	
	No	2737	89.6	
In family	Yes	551	18.0	
	No	2505	82.0	
In caring for others	Yes	484	15.9	
	No	2562	84.1	
**Presence of any illness or health problem**	Yes	1115	36.5	
	No	1945	63.5	
**Health status**	Excellent	253	8.3	
	Very good	462	15.1	
	Good	1476	48.2	
	Fair	753	24.6	
	Poor	115	3.8	

### Data collection

Data was collected using a questionnaire with three main parts. The first section comprised demographic questions about age, gender, education level, employment status, and marital status. These questions were according to the demographic part of a questionnaire provided by the Statistical Center of Iran (SCI), for the national population and housing census [14]. The second part was regarding the general health status questions about the respondent’s viewpoints of his/her health and the presence of any illness or health problem in respondents or their family members. Health status was measured by a categorical measure. In the categorical health rating question, individuals rated their own current health status on a 5-point scale (excellent, very good, good, fair, or poor). The presence of any illness or health problem was assessed with the question: “Do you have any illness, health problem, condition, or disability?”. The third part consisted of the official Iranian version of the EQ-5D-5 L. The EQ-5D-5 L consists of two pages: the descriptive system and the Visual Analogue Scale (EQ-VAS). The EQ-5D-5 L descriptive system consists of five dimensions as follows: mobility (MO), self-care (SC), usual activities (UA), pain/discomfort (PD), and anxiety/ depression (AD). Each dimension in the EQ-5D-5 L has five response levels: no problems (Level 1); slight; moderate; severe; and extreme problems (Level 5). Total 3125 (55) health states are defined for EQ-5D-5 L. Health states are from 11,111 (the best health state) to 55,555 (the worst health state). EQ-5D-5 L health states are converted into a single index ‘utility’ score using a scoring algorithm based on public preferences. The instrument also includes a visual analogue scale (EQ-VAS) which provides a single global rating of self-perceived health and is scored on a 0 to 100 mm scale representing “the worst …” and “the best health you can imagine”, respectively.

### Data analysis

To measure the respondents’ HRQoL scores according to the EQ-5D-5 L questionnaire, since a standard EQ-5D-5 L value set was not available for Iran, the five-level crosswalk-based value set derived from the EQ-5D-3 L value set in Iran was used [13, 15]. A crosswalk-based value set is an interim scoring method for the EQ-5D-5 L that allows EQ-5D-5 L values to be derived from any existing EQ-5D-3 L value set. The crosswalk is based on a response mapping approach that estimates the relationship between responses to the EQ-5D-3 L (‘3 L’) and EQ-5D-5 L (‘5 L’) descriptive systems, and subsequently establishes a link to the established 3 L value sets [16]. The crosswalk methodology developed by van Hout et al. [16], was applied to the Iran EQ-5D-3 L value set [13] developed using a face-to-face TTO method to obtain the Iran crosswalk EQ-5D-5 L value set.

Descriptive summary statistics were estimated for sociodemographic variables, the EQ-5D-5 L dimensions, utility scores, and self-reported health status. The distribution of answers to the questions in the descriptive part of the EQ-5D-5 L was estimated for the whole sample, as well as for the different age groups. To assess the mean EQ-5D-5 L score differences between socio-demographic groups, the Wilcoxon rank-sum test (for variables with two sub-groups) and the Kruskal-Wallis test (for variables with multiple sub-groups) were applied [17, 18].

The Generalized linear model (GLM) was employed to explore (understand) the association between socio-demographic variables and EQ-5D utility scores (model 1) as well as VAS scores (model 2). This model is able to control skewness and heteroscedasticity. We used GLM model with a Poisson distribution and a log link, which requires none-negative values. Therefore disutility value (disutility = 1-utility value) was entered as the dependent variable [17, 19]. The analyses were conducted using STATA version 13.

## Results

Table 1 shows the descriptive statistics of the variables. The response rate was about 70%. In the current study, 51% of the participants were female; the mean ± standard deviation (SD) of the participants’ age was 44 ± 15.6 years; about 63% of the participants were younger than 50 years old; and the average education years was 10.8 ± 4.8. In other words, 4.6% of the participants were illiterate, about 70% had high school diploma or lower, and 30% had a university education. Most of the participants were homemaker (40.5%); 35.5% were unemployed, but had income, and 7.6% were students; 77.3% of the subjects were married (had spouse), 18% were single (never married), and 4.7% were divorced or widowed.

Approximately, 10.4% of the participants experienced a serious illness, and 36.5% of them had illness or health problem. The results of self-rated health status show that 23% of the participants’ health status was “excellent” or “very good”, while 4% was “poor”.

The health status of the participants according to the dimensions of the EQ-5D-5 L questionnaire is reported in the Additional file 1. About 70.5% of the individuals had no problems in walking, while 0.1% were unable to walk; 90.6% of the participants had no problems washing or dressing himself/herself, while 0.1% were unable; 76.3% of the individuals had no problems doing his/her usual activities, while 0.3% were unable; 46.7% of the participants had no pain or discomfort, while 0. 8% had extreme pain or discomfort. Considering the “anxiety/depression”, 46% of the individuals stated no anxiety or depression, while 2.1% were extremely anxious or depressed.

**Fig. 1:**
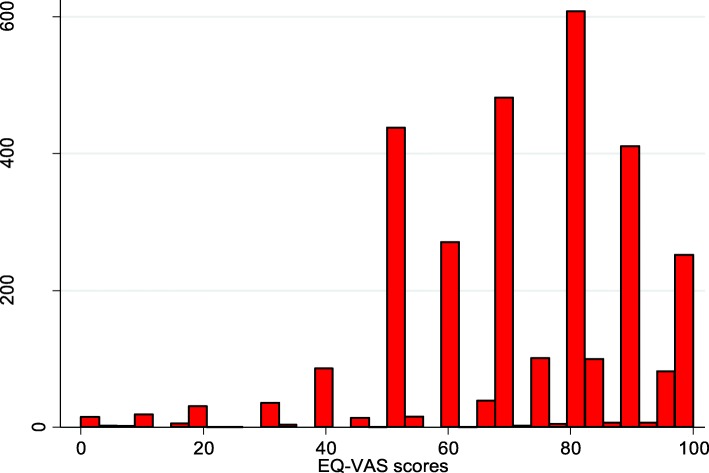
**a** Distribution of the health-related quality of life Scores, **b** Distribution of EQ-VAS Scores

Figure 1 shows the participants’ HRQoL (utilities) and EQ-VAS scores. The HRQoL scores were left-skewed. They ranged from 0.013 to 1. The mean HRQoL score was 0.79 ± 0.17. The EQ-VAS scores ranged from 0 to 100. The mean EQ-VAS score was 71.7 ± 19.4.

Table 2 shows the mean EQ-5D-5 L utility scores by sociodemographic characteristics. The mean utility scores range 0.61 ± 0.19 in > 69 year-old females to 0.88 ± 0.12 in < 30 year-old males. Table 3 shows the mean EQ-VAS scores by gender and age groups. The mean VAS scores range 57.5 ± 23.6 in > 69 year-old females to 80 ± 16.3 in < 30 year-old males.

**Table 2:** Mean EQ-5D-5 L utility scores by sociodemographic characteristics

Variable	Total			Males			Females		
	number	Mean	Std.Dev.	number	Mean	Std.Dev.	Number	Mean	Std.Dev.
All	3060	0.79	0.17	1505	0.83	0.16	1555	0.76	0.17
Z = 11.473,*p* < 0.001									
**Age category, years**									
< 30	609	0.87	0.12	341	0.88	0.12	268	0.86	0.12
30–39	726	0.83	0.15	342	0.85	0.14	384	0.81	0.15
40–49	602	0.78	0.15	252	0.83	0.14	350	0.75	0.15
50–59	539	0.75	0.17	234	0.81	0.16	305	0.71	0.16
60–69	389	0.74	0.18	207	0.79	0.18	182	0.68	0.17
> 69	195	0.67	0.20	129	0.70	0.20	66	0.61	0.19
Total		*X*^*2*^ = 291.367, *p* < 0.001							
**Education level**									
Illiterate	140	0.68	0.22	52	0.73	0.23	88	0.64	0.20
Elementary	387	0.73	0.18	141	0.76	0.20	246	0.71	0.17
Secondary	516	0.76	0.17	256	0.81	0.16	260	0.72	0.16
High	1100	0.81	0.15	507	0.84	0.15	593	0.78	0.15
University	917	0.84	0.14	549	0.86	0.14	368	0.82	0.14
Total		*X*^*2*^ = 172.145, *p* < 0.001							
**Employment status**									
Employed	1079	0.84	0.14	936	0.85	0.14	143	0.79	0.15
Student	221	0.88	0.11	135	0.89	0.10	86	0.87	0.13
Homemaker	1239	0.75	0.17	4	0.72	0.27	1235	0.75	0.17
Retired	395	0.76	0.18	341	0.76	0.18	54	0.76	0.18
Unemployed	100	0.82	0.17	66	0.79	0.18	34	0.88	0.11
Other	22	0.71	0.26	21	0.71	0.27	1	0.63	0.00
Total		*X*^*2*^ = 251.442, *p* < 0.001							
**Marital status**									
Never married	549	0.87	0.13	387	0.87	0.12	162	0.86	0.13
Married	2358	0.78	0.17	1082	0.81	0.17	1276	0.76	0.16
Divorce or widowed	144	0.67	0.17	27	0.71	0.16	117	0.66	0.17
Total		*X*^*2*^ = 178.314, *p* < 0.001							

Wilcoxon rank-sum test and Kruskal-Wallis test run for EQ-5D sum scores

**Table 3:** Mean EQ-VAS Scores by gender and age

Variable	Total			Males			Females		
	number	Mean	Std.Dev.	number	Mean	Std.Dev.	number	Mean	Std.Dev.
All	3042	71.7	19.37	1498	73.77	18.67	1544	69.74	19.83
Z = 5.696, *p* < 0.001									
**Age category, years**									
< 30	607	79.9	15.8	340	80.0	16.3	267	79.8	15.1
30–39	723	75.1	17.1	341	76.1	16.5	382	74.2	17.6
40–49	599	70.2	17.7	252	73.0	16.5	347	68.2	18.3
50–59	535	66.8	20.9	232	70.6	20.3	303	63.8	20.9
60–69	386	66.7	20. 8	205	70.2	20.1	181	62.6	20.9
> 69	192	61. 8	22.5	128	63.9	21.7	64	57.5	23.6
Total		*X*^*2*^ = 242.371, *p* < 0.001							

Wilcoxon rank-sum test and Kruskal-Wallis test run for VAS scores

Men had higher scores than women. The average score of men was 0.83 ± 0.16 while the average score of women was 0.76 ± 0.17. The mean VAS score was 73 ± 18 for men versus 69 ± 19 for women. Both EQ-5D and EQ-VAS scores reduced with age increase. The EQ-5D utility scores shows significant difference in all groups of socio-demographic characteristics, including educational level employment status and marital status.

Table 4 shows the results of the regression model. In the first model the relationships were not significant about most of the factors. Although, in the second model, there was a significant relationship between gender and the VAS scores; on average, scores were lower in females than males. The VAS scores reduced with age increase; the higher educational level led to higher scores. The VAS scores among individuals without spouse (divorced or widowed) were significantly lower than those of the married individuals or the never married ones. The VAS scores were lower in homemakers than the others.

**Table 4:** GLM poisson regression to determine the effective factors on the health related quality of life disutility scores and EQ-VAS scores

Independent variable	Dependent variable: disutility scores			Dependent variable: EQ-VAS scores		
	Model 1			Model 2		
	Coefficient	***p***-value	95% CI	Coefficient	***p***-value	95% CI
**Gender**						
Male	Ref					
Female	0.215	0.164	− 0.088,0.519	−0.014	0.054	−0.028,0.000
**Age category**						
< 30	Ref					
30–39	0.226	0.178	−0.103,0.555	− 0.050	0.000	−0.064,-0.035
40–49	0.416	0.016	0.076,0.756	−0.106	0.000	−0.122,-0.090
50–59	0.494	0.006	0.143,0.844	−0.139	0.000	−0.156,-0.122
60–69	0.547	0.005	0.161,0.932	−0.144	0.000	−0.164,-0.124
> 69	0.785	0.001	0.334,1.236	−0.208	0.000	−0.235,-0.182
**Marital status**						
Never married	Ref					
Married	0.053	0.749	−0.275,0.383	0.014	0.046	0.000,0.029
Divorced or widowed	0.223	0.338	−0.233,0.679	−0.107	0.000	−0.134,-0.080
**Employment status**						
Employed	Ref					
Student	−0.032	0.897	−0.526,0.461	0.009	0.355	−0.010,0.028
Home maker	0.149	0.381	−0.185,0.484	− 0.039	0.000	−0.055,-0.022
Retired	0.093	0.553	− 0.214,0.400	0.007	0.397	−0.009,0.025
Unemployed	0.152	0.563	−0.364,0.670	0.007	0.545	−0.017,0.032
Others	0.569	0.164	−0.232,1.372	−0.213	0.000	−0.268,-0.0158
**Education**						
Illiterate	Ref					
Elementary	−0.010	0.957	−0.377,0.357	0.017	0.192	−0.008,0.043
Guidance	−0.073	0.692	− 0.436,0.290	0.042	0.001	0.017,0.067
High school and pre-university	−0.159	0.386	−0.519,0.200	0.078	0.000	0.053,0.102
University	−0.208	0.293	−0.598,0.180	0.094	0.000	0.068,0.120
Constant	−2.115	0.000	−2.607,-1.622	4.307	0.000	4.279,4.335
AIC	0.899			11.590		
BIC	−23,823.73			− 7364.766		

## Discussion

The current study provided the EQ-5D-5 L utility scores and EQ-VAS and their determinants among the adult population of Tehran. About 71, 91, 76, 47, and 46% of the participants reported no problems on mobility, self-care, usual activities, pain/discomfort, and anxiety/depression, respectively. The HRQoL mean score was 0.79 ± 0.17 based on crosswalk method. The VAS mean score was 71.7 ± 19.4. Considering EQ-VAS scores gender, age, education status, marital status, and employment status were associated withHRQoL.

In a previous study by Goudarzi et al. in Iran, using the three level questionnaire, the percentages of individuals reporting no problems on mobility, self-care, usual activities, pain/discomfort, and anxiety/depression were 89, 99, 96, 66, and 67%, respectively [13]. These figures were measured as 71, 91, 76, 47, and 46% in the present study, respectively. The ratios were very similar in each dimension. For example, in both studies the majority of people had no problems in self-care. Moreover, individuals had problems with anxiety/depression, and pain/discomfort more than the other dimensions. However, in the current study, the percentage of individuals with “no problems” was less than that of the previous study in all dimensions. It might be due to the higher sensitivity and the lower “ceiling effect” of the EQ-5D-5 L compared with the EQ-5D-3 L questionnaire [20–22].

The Iranian participants reporting no problems were lower than those of some other countries such as South Australia, Poland, Italy, and Germany. This issue was observed in all the dimensions, which was notably prominent in “anxiety/depression” dimension. The percentage of individuals with no anxiety or depression was 46% in Iran, which was 73.3, 58.5, 61.7 and 77.4% in South Australia, Poland, Italy, and Germany, respectively [11, 12, 17, 23].

It is interesting to note that in anxiety/depression and pain/discomfort dimensions the individuals mainly had moderate problems, which was true in all age groups. While in other dimensions, more than half of the sample had no problems in lower age groups and the percentage of individuals with problems increased in higher age groups. It was consistent with the finding of other countries [11, 12, 17]. High percentage of the anxiety/depression in all ages is a sign that highlights the risk of the psychological disorders, which requires purposeful considerations and measures.

The mean utility score of the current study participants (0.79 ± 0.17) seems lower than those of Germany (0.92) [23], South Australia (0.91) [17], Poland (0.89) [11], Uruguay (0.95) [24], and Italy (0.92) [12]; and was very similar to the previously reported value (0.79) using the three level version in an adult sample of Tehran [15]. This likeness arise from the studies’ population and value set similarities.

The study showed that females had lower utility scores than males. Goudarzi et al., indicated that in all dimensions, females had more problems than males [13], which was also confirmed by other studies [25, 26].

The regression analyses represented that male, younger, and more educated individuals were more probable to have a better EQ-VAS scores, which coincided with the findings of other countries [17, 27, 28].

The VAS score of single subjects (divorced or widowed) was significantly lower than those of the married or never married ones. Recently, the divorce rate increased in Iran. The marriage to divorce ratio was 16 in 1993, which reduced to 4.4 in 2014 [29]. Due to the problems that families might face, in addition to the families’ HRQoL reduction due to divorce or the spouse death [30], effective interventions are needed to strengthen the families, reduce divorce, and support such vulnerable groups.

The current study for the first time applied the EQ-5D-5 L in a large sample in Tehran. Tehran is a highly populated city (consisting 11% of Iran population) and consists of different ethnic groups, which can be a proper representative for Iran community.

## Conclusion

The current study provided HRQoL scores and their determinants for the Iranian adult population, which was applicable for the policy makers. In fact, having an accurate perspective of the society health status helps the planners and policy makers in decision making. Since more than half of the subjects described their health status moderately or extremely anxious or depressed, it is recommended that more attention be paid to the spiritual morbidities and effective intervention be implemented to prevent such diseases. Due to the relatively low utility scores of the Iranians, long term planning is required to increase their health scores, especially for the vulnerable groups of the community.

## Supplementary information

**Additional file 1.**

## Data Availability

The datasets used and/or analyzed during the current study are available from the corresponding author on reasonable request.

## Abbreviations

HRQoL: Health-Related Quality of Life
QoL: Quality of Life
VAS: Visual Analog Scale
