# Correction to: Health-related quality of life measured using the EQ-5D-5 L: population norms for the capital of Iran

**DOI:** 10.1186/s12955-020-01397-x

**Published:** 2020-05-21

**Authors:** Zahra Emrani, Ali Akbari Sari, Hojjat Zeraati, Alireza Olyaeemanesh, Rajabali Daroudi

**Affiliations:** 1 Department of Health Management and Economics, School of Public Health, Tehran University of Medical Sciences, Poursina Ave, Tehran, 1417613191 Iran; 2 Department of Epidemiology and Biostatistics, Tehran University of Medical Sciences, Tehran, Iran; 3 National Institute for Health Research & Health Equity Research Centre, Tehran University of Medical Sciences, Tehran, Iran; 4 Department of Health Management and Economics, School of Public Health, Tehran University of Medical Sciences, Poursina Ave, Tehran, 1417613191 Iran

**Correction to: Health Qual Life Outcomes (2020) 18:108**

**https://doi.org/10.1186/s12955-020-01365-5**

The original article [1] contained an error mistakenly carried forward by the production team handling this article whereby sub-panel ‘a’ from Fig. 1 was omitted from published article. This has since been corrected.
