# Supplementary material for: Health-related quality of life measured using the EQ-5D–5 L: population norms for the capital of Iran
Source: Health Qual Life Outcomes. 2020 Apr 25;18:108. doi: 10.1186/s12955-020-01365-5 (PMC7183694; doi:10.1186/s12955-020-01365-5)
Supplement: Supplementary file 1 — Additional file 1. [file 12955_2020_1365_MOESM1_ESM.docx]

Table S1: Frequency of Item Responses in Each EQ-5D-5L Dimension by Age

|  |  |  |  | Age category, years | | | | |  |  |  |  |  |  |
| --- | --- | --- | --- | --- | --- | --- | --- | --- | --- | --- | --- | --- | --- | --- |
| Dimension | <30 | | 30-39 | | 40-49 | | 50-59 | | 60-69 | | >69 | | Total | |
|  | n | % | n | % | n | % | n | % | n | % | n | % | n | % |
| **Mobility** |  |  |  |  |  |  |  |  |  |  |  |  |  |  |
| No problems | 554 | 90.9 | 588 | 81.0 | 418 | 69.4 | 320 | 59.4 | 208 | 53.3 | 69 | 35.3 | 2,157 | 70.5 |
| Slight problems | 41 | 6.7 | 91 | 12.5 | 112 | 18.6 | 116 | 21.5 | 88 | 22.6 | 43 | 22 | 491 | 16.0 |
| Moderate problems | 12 | 1.9 | 36 | 5.0 | 53 | 8.8 | 76 | 14.1 | 59 | 15.1 | 51 | 26.1 | 287 | 9.4 |
| Severe problems | 2 | 0.0 | 11 | 1.5 | 19 | 3.2 | 26 | 4.8 | 35 | 9.0 | 31 | 15.8 | 124 | 4.1 |
| incapacity | 0 | 0.3 | 0 | 0.0 | 0 | 0.0 | 1 | 0.2 | 0 | 0.0 | 1 | 0.5 | 2 | 0.1 |
| **Self-care** |  |  |  |  |  |  |  |  |  |  |  |  |  |  |
| No problems | 595 | 97.7 | 695 | 95.7 | 563 | 93.5 | 470 | 87.2 | 323 | 82.8 | 128 | 65.6 | 2,774 | 90.6 |
| Slight problems | 10 | 1.6 | 24 | 3.3 | 27 | 4.5 | 42 | 7.8 | 45 | 11.5 | 38 | 19.4 | 186 | 6.1 |
| Moderate problems | 4 | 0.6 | 7 | 1.0 | 8 | 1.3 | 23 | 4.3 | 15 | 3.9 | 22 | 11.2 | 79 | 2.6 |
| Severe problems | 0 | 0.0 | 0 | 0.0 | 4 | 0.7 | 4 | 0.7 | 6 | 1.5 | 5 | 2.5 | 19 | 0.6 |
| incapacity | 0 | 0.0 | 0 | 0.0 | 0 | 0.0 | 0 | 0.0 | 1 | 0.3 | 2 | 1 | 3 | 0.1 |
| **Usual activities** |  |  |  |  |  |  |  |  |  |  |  |  |  |  |
| No problems | 558 | 91.6 | 609 | 83.9 | 468 | 77.7 | 370 | 68.7 | 241 | 62.0 | 90 | 46.1 | 2,336 | 76.3 |
| Slight problems | 38 | 6.2 | 76 | 10.5 | 83 | 13.8 | 101 | 18.7 | 86 | 22.1 | 45 | 23 | 429 | 14.0 |
| Moderate problems | 11 | 1.8 | 30 | 4.1 | 46 | 7.6 | 56 | 10.4 | 47 | 12.1 | 46 | 23.5 | 236 | 7.7 |
| Severe problems | 2 | 0.3 | 10 | 1.4 | 5 | 0.8 | 10 | 1.9 | 12 | 3.1 | 12 | 6.1 | 51 | 1.7 |
| incapacity | 0 | 0.0 | 1 | 0.1 | 0 | 0.0 | 2 | 0.4 | 3 | 0.8 | 2 | 1 | 8 | 0.3 |
| **Pain/discomfort** |  |  |  |  |  |  |  |  |  |  |  |  |  |  |
| No | 434 | 71.2 | 387 | 53.3 | 243 | 40.4 | 195 | 36.2 | 125 | 32.1 | 47 | 24.1 | 1,431 | 46.8 |
| Slight | 121 | 19.8 | 212 | 29.2 | 194 | 32.2 | 170 | 31.5 | 117 | 30.1 | 42 | 21.5 | 856 | 28.0 |
| Moderate | 45 | 7.3 | 91 | 12.5 | 124 | 20.6 | 110 | 20.4 | 101 | 26.0 | 72 | 36.9 | 543 | 17.8 |
| Severe | 9 | 1.4 | 32 | 4.4 | 40 | 6.6 | 58 | 10.8 | 38 | 9.8 | 29 | 14.8 | 206 | 6.7 |
| Extreme | 0 | 0.0 | 4 | 0.6 | 1 | 0.2 | 6 | 1.1 | 8 | 2.1 | 5 | 2.5 | 24 | 0.8 |
| **Anxiety/depression** |  |  |  |  |  |  |  |  |  |  |  |  |  |  |
| No | 332 | 54.7 | 345 | 47.5 | 240 | 39.9 | 227 | 42.1 | 185 | 47.4 | 78 | 40 | 1,407 | 46.0 |
| Slight | 164 | 26.9 | 202 | 27.8 | 165 | 27.4 | 141 | 26.2 | 98 | 25.1 | 43 | 22 | 813 | 26.6 |
| Moderate | 85 | 13.9 | 132 | 18.2 | 138 | 22.9 | 108 | 20.0 | 71 | 18.2 | 56 | 28.7 | 590 | 19.3 |
| Severe | 16 | 2.6 | 37 | 5.1 | 40 | 6.6 | 52 | 9.7 | 26 | 6.7 | 16 | 8.2 | 187 | 6.1 |
| Extreme | 12 | 1.9 | 10 | 1.4 | 19 | 3.2 | 11 | 2.0 | 10 | 2.6 | 2 | 1 | 64 | 2.1 |
